# Gan-Jiang-Ling-Zhu Decoction Prevents Paigen’s Diet-induced Lean Metabolic Dysfunction-associated Steatotic Liver Disease by Regulating Bile Acid Metabolism

**DOI:** 10.2174/0113862073379080250701164923

**Published:** 2025-07-15

**Authors:** Zansong Ma, Milian Chen, Ying Cao, Deji Song, Li Zhang

**Affiliations:** 1 Institute of Digestive Diseases, Longhua Hospital, Shanghai University of Traditional Chinese Medicine, Shanghai 200032, China;; 2 Fifth People’s Hospital, Fudan University, Shanghai 201100, China;; 3 State Key Laboratory of Integration and Innovation of Classical Formula and Modern Chinese Medicine, Shanghai, China

**Keywords:** Chinese traditional medicine, gan-Jiang-Ling-zhu decoction, lean non-alcoholic fatty liver disease (MASLD), gut microbiota, bile acid, hepatic steatosis

## Abstract

**Introduction:**

Metabolic dysfunction-associated steatotic liver disease (MASLD) is a global health concern, even among lean individuals. The Gan-Jiang-Ling-Zhu decoction (GZD), a traditional Chinese medicine formula, shows therapeutic potential against MASLD. This study investigated the efficacy of GZD in lean MASLD and explored its mechanisms of action.

**Methods:**

A lean MASLD mouse model was established using C57BL/6 mice fed with a cholesterol-rich Paigen’s diet (PD). Following successful modeling, mice were administered GZD (1.8, 3.6, or 7.2 g/kg) or vehicle control. Body weight, food intake, and liver weight were monitored. Hepatic steatosis and lipid accumulation were assessed *via* H&E and Oil Red O staining, while serum enzymes were quantified biochemically. Gut microbiota composition was analyzed by 16S rRNA gene sequencing, and bile acid (BA) profiles in feces and serum were measured using UPLC-TQMS.

**Results:**

Twelve weeks of PD feeding induced a lean MASLD phenotype characterized by reduced body weight alongside hepatic steatosis and dyslipidemia. The GZD treatment dose-dependently ameliorated liver steatosis and lipid accumulation, with the highest dose (7.2 g/kg) showing superior efficacy. GZD restored gut microbiota balance by reducing pathogenic bacteria and enriching taxa involved in BA metabolism, leading to increased fecal excretion of secondary BAs. Conversely, serum levels of secondary BAs were significantly reduced after GZD treatment.

**Discussion:**

Our study highlights the promising role of GZD in lean MASLD, the involvement of gut microbiota and related BA metabolism that aligns with emerging evidence that gut dysbiosis and disrupted BA homeostasis are central to MASLD pathogenesis, even in lean individuals. However, the mechanistic links between specific microbial changes, BA pool composition, and hepatic outcomes remain to be elucidated.

**Conclusion:**

GZD ameliorates hepatic steatosis in lean MASLD mice, an effect associated with modulation of gut microbiota composition and increased fecal excretion of secondary BAs. These findings suggest the potential of GZD as a therapeutic option for lean MASLD through gut-liver axis regulation.

## 1 INTRODUCTION

Metabolic Dysfunction-Associated Steatotic Liver Disease (MASLD), formerly termed Non-Alcoholic Fatty Liver Disease (NAFLD), is characterized by hepatic steatosis concurrent with metabolic dysregulation, including insulin resistance, dyslipidemia, and central adiposity [1]. MASLD has emerged as a global epidemic, affecting over 30% of the adult population worldwide [2]. While obesity remains a predominant risk factor, a clinically significant subset of MASLD manifests in lean individuals, defined by a body mass index (BMI) <25 kg/m² in Western populations and <23 kg/m² in Asian populations, termed lean MASLD [3]. Epidemiological studies estimate the prevalence of lean MASLD at 7%-23% of the global MASLD population, with notably higher rates observed in Asian countries compared to Western regions [4-6]. Despite their lower adiposity, lean MASLD patients exhibit a metabolically unhealthy phenotype, marked by insulin resistance, dyslipidemia, and cardiovascular risk profiles comparable to those observed in obese MASLD cohorts [7]. Notably, emerging evidence suggests that lean MASLD may confer an elevated risk of disease progression, including hepatic fibrosis and cirrhosis, compared to obese counterparts [8, 9]. Current therapeutic options for MASLD remain limited. While Resmetirom has recently gained regulatory approval as the first pharmacotherapy for metabolic dysfunction-associated steatohepatitis (MASH) due to its demonstrated efficacy in addressing MASH with concomitant fibrosis, no targeted pharmacotherapy exists for lean MASLD [10, 11]. This unmet clinical need underscores the urgency for mechanistic investigations and the development of phenotype-specific therapeutic strategies.

Emerging evidence highlights dietary cholesterol as a key driver of lean MASLD pathogenesis [12]. Clinical studies associate high cholesterol intake with lean MASLD incidence independent of weight gain [13], while animal models demonstrate that cholesterol-enriched diets, such as the Paigen Diet (PD), recapitulate hepatic steatosis, dyslipidemia, and paradoxical weight loss, a hallmark of human lean MASLD [14-16]. This phenotype coincides with disrupted bile acid (BA) metabolism, as patients with lean MASLD exhibit distinct BA profiles characterized by elevated toxic secondary BAs (*e.g.*, deoxycholic acid) and reduced farnesoid X Receptor (FXR) signaling, implicating gut-liver axis dysregulation in disease progression [17]. Given that BAs are both cholesterol derivatives and modulators of gut microbiota composition, these findings suggest a pathogenic interplay between cholesterol homeostasis, BA dysmetabolism, and microbial dysbiosis in lean MASLD-a nexus that remains underexplored therapeutically.

Traditional Chinese Medicine (TCM) offers promising multi-target approaches for metabolic disorders. Gan-Jiang-Ling-Zhu decoction (GZD) is a classical formula derived from the legend of Traditional Chinese medicine “Jin-Kui-Yao Lue”. It is composed of *Glycyrrhiza uralensis Fisch* (Gancao), *Zingiber officinale Rosc* (Ganjiang), *Poria cocos (Schw.) Wolf* (Fuling), and *Atractylodes macrocephala* (Baizhu). Prior studies have identified hepatoprotective properties in bioactive components of GZD-such as pachydermic acid (anti-inflammatory) [18],  pachyman (immunomodulatory) [19], and Atractylenolides （I,II,III）[20] are reported to obtain direct hepatoprotective properties and counteract liver injuries in animal experiments. Preclinical studies demonstrate that GZD reduces hepatic steatosis by enhancing hepatic CPT1B-mediated fatty acid oxidation, while concurrently ameliorating gut dysbiosis and modulating protein methylation processes implicated in metabolic regulation [21-23]. However, its mechanisms, particularly its impact on cholesterol-BA-microbiota crosstalk, remain unknown. Here, we investigated GZD’s therapeutic potential in a PD-induced lean MASLD murine model. We demonstrate that GZD alleviates hepatic steatosis, dyslipidemia, and gut dysbiosis while normalizing toxic BA accumulation. Our findings position GZD as a novel candidate for lean MASLD treatment, mechanistically linked to restoration of cholesterol-BA homeostasis and microbial ecology.

## 2 METHODS

### 2.1 Preparation of GZD

GZD is composed of *Glycyrrhiza uralensis Fisch* (Gancao), *Zingiber officinale Rosc* (Ganjiang) , *Poria cocos (Schw.) Wolf* (Fuling), and *Atractylodes macrocephala* (Baizhu) with a ratio of 1:2:2:1. All herbs and the decoction were prepared by the Pharmacy department, Longhua Hospital affiliated to Shanghai University of TCM, and quality control was conducted as previously reported [21].

### 2.2 Animals and Experimental Design

It is an experimental study that adheres to internationally accepted standards for animal research, following the 3Rs principle by using the minimum number of animals required to detect significant effects. The ARRIVE guidelines were employed for reporting experiments involving live animals, promoting ethical research practices. The animal experiments were approved by the Animal Experiment Ethics Committee of the Shanghai University of Traditional Chinese Medicine (PZSHUTCM2305100003). Male C57Bl/6J mice (8 weeks old, body weight 20-22g) were purchased from Gempharmatech Experimental Animal Technology Co., Ltd. (Jiangsu, China), and housed in a temperature (20-22°C)- and humidity (50%-60%)-controlled specific-pathogen-free environment. The mice were given free access to water and food, and their body weights were recorded weekly.

Following a 1-week acclimation period, mice were allocated into three dietary groups (*n*=6 per group): a standard chow diet (Con), a Paigen Diet (PD; 40% fat, 1.25% cholesterol, 0.45% sodium cholate; Research Diets, D12109C), or a Western Diet (WD; Harlan, TD.88137), administered for 12 weeks. The human clinical dose of GZD (36 g/kg/day) was converted to a mouse equivalent dose (medium) using body surface area normalization (Reagan-Shaw formula). Lean MASLD mice were randomized into four groups following successful model validation based on the body weight: (**1**) vehicle control (normal saline, NS), (**2**) low-dose GZD (GZD-L, 1.8 g/kg/day), (**3**) medium-dose GZD (GZD-M, 3.6g/kg/day), and (**4**) high-dose GZD (GZD-H, 7.2 g/kg/day). Treatments were administered daily *via* oral gavage for 4 weeks. At the end of each experiment, the mice were anesthetized, followed by collecting the blood samples, feces, and liver tissues. All of the samples were then frozen at −80°C until use.

### 2.3 Histological examination of liver tissues

Liver tissues were harvested, fixed in 4% formaldehyde for 24 hours, and processed with the paraffin sectioning procedure. Then, the paraffin-embedded liver sections (4 µm) were stained with hematoxylin and eosin (H&E) according to the established protocol, whereas the frozen sections (8 µm) were stained with oil red O (ORO) solution [24].

### 2.4 Analysis of Serum Samples

Serum alanine aminotransferase (ALT), aspartate aminotransferase (AST), triglyceride (TG), total cholesterol (TC), high-density lipoprotein cholesterol (HDL-c), low-density lipoprotein cholesterol (LDL-c), alkaline phosphatase (ALP) and total BA (TBA) levels were detected by a TBA-40FR automatic biochemical analyzer (Toshiba, Japan) according to the testing procedures of manufacturers.

### 2.5 RT‑qPCR Analysis

Total RNA from liver tissue was extracted using TRIzol reagent (Thermo Fisher Scientific, USA), and a NanoDrop spectrophotometer (2000c, NanoDrop technology, USA) was used to determine the RNA concentration. Following reverse transcription to cDNA, PCR was performed on a QuantStudio 6 Real-Time PCR System (Thermo Fisher Scientific) using the SYBR Green kit (Accurate Biology, AG11701). β-actin was used as a reference gene to standardize mRNA expression levels, and 2^-ΔΔCt^ was used to analyze relative mRNA expression. The sequences of primers (Shanghai Shanjin Biotechnology, China) are shown in Table **1**.

### 2.6 Gut Microbiota Analysis

The gut microbiota was analyzed by 16S rDNA sequencing following a previously published method [25]. In brief, fecal and serum samples were collected from each mouse and stored at -80°C. Genomic DNA was extracted from fecal samples using the QIAamp DNA Stool Kit (Qiagen, Germany), and the purity and concentration of DNA were determined *via* a NanoDrop ND-2000C spectrophotometer (Thermo Fisher Scientific, USA) and agarose gel electrophoresis. The V3-V4 region of the bacterial 16S ribosomal ribonucleic acid (rRNA) gene was amplified using polymerase chain reaction (PCR); sample-specific 7-bp barcodes were incorporated into the primers for multiplex sequencing. PCR amplicons were purified with Vazyme VAHTSTM DNA Clean Beads (Vazyme, Nanjing, China) and quantified using the Quant-iT PicoGreen dsDNA Assay Kit (Invitrogen, Carlsbad, CA, USA). After the individual quantification step, amplicons were pooled in equal amounts, and paired-end 2×250 bp sequencing was performed using the Illumina NovaSeq platform with a NovaSeq 6000 SP Reagent Kit (500 cycles). The sequences clustered with 97% similar taxonomy were grouped as operational taxonomic units (OTUs). The free online Genetic Cloud Platform (Personal Biotechnology Co., Ltd., Shanghai, China) was used to conduct bioinformatics and statistical analysis.

### 2.7 Detection of the BA Proﬁle

Fecal and serum BA components were quantified using ultra-performance liquid chromatography coupled with triple quadrupole mass spectrometry (UPLC-TQMS, Waters, Milford, MA) according to a previously established protocol [26, 27]. Briefly, 20 mg of each fecal sample was weighed and homogenized with 200 μL methanol/acetonitrile (*v/v*=2:8, with internal standards each 50 nM) after the samples were ground with a ball mill. After this, 50 μL of each serum sample was extracted with the same solution. The samples were then kept at 20°C for 10 min to precipitate proteins, followed by centrifugation for 10 min (12000 r/min, and 4°C). The supernatant was then seperated and the extracts were evaporated to dryness, and reconstituted in 100 uL 50% methanol (*v/v*) for further analysis.

### 2.8 Statistical Analysis

SPSS software (version 26.0; IBM, Armonk, NY, USA) was used to analyze all the data, and the data are presented as the means *±* standard deviations. Two-tailed Student’s t-test was utilized to analyze the differences between the two groups, and one-way analysis of variance was utilized, followed by Dunnett’s post hoc tests to confirm the differences among groups. *P-value less than* 0.05 was considered significant. GraphPad Prism version 8.0.2 (San Diego, CA, USA) was employed to prepare the graphs.

## 3 RESULTS

### 3.1 PD induces a Lean MASLD Phenotype in Mice

The Paigen Diet (PD), a well-characterized regimen for inducing atherosclerosis models, has recently been demonstrated to elicit lean MASLD. To evaluate its efficacy in this context, we conducted a comparative analysis between PD (40% fat, 1.25% cholesterol, 0.45% sodium cholate; Research Diets, D12109C) and the Western Diet (WD; Harlan, TD.88137), a widely recognized dietary model for MASLD induction, by administering both diets to C57BL/6J mice over a 12-week experimental period (Fig. **1A**). The dynamic records showed divergent trends between dietary groups. WD-fed mice demonstrated a steady increase in body weight from week 8 onward. In contrast, mice fed with the PD exhibited a progressive decline in body weight commencing at week 8 post-diet initiation, with statistically significant reductions observed by week 9 (Fig. **1B**). Despite these opposing weight trajectories, both PD- and WD-fed mice displayed significant hepatomegaly at the study endpoint, as indicated by elevated liver-to-body weight ratios (Fig. **1C**). The histopathological analysis further confirmed marked hepatic steatosis and lipid accumulation in these groups, evidenced by H&E staining and ORO staining of liver sections (Fig. **1D**). Notably, serum ALT and AST levels remained within normal physiological ranges across all groups, suggesting preserved hepatic function despite pronounced steatosis (Figs. **1E** and **F**). The serum lipid profiling revealed profound dyslipidemia in PD-fed mice, characterized by elevated TG, TC, HDL-c, and LDL-c levels comparable to those in WD-fed mice (Figs. **1G**-**J**). Collectively, these findings demonstrate that 12-week PD administration recapitulates key features of lean MASLD, including steatosis, hepatomegaly, and dyslipidemia, without body weight increase in mice.

### 3.2 GZD Improves Hepatic Steatosis in Lean MASLD Mice

To assess the therapeutic potential of GZD, lean-MASLD mice were administered three escalating doses of GZD (low, medium, high) *via* oral gavage (Fig. **2A**). PD intake remained comparable across all treatment groups during the study, confirming uniform dietary exposure (Fig. **2B**). While GZD exerted no significant effect on final body weight, it dose-dependently reduced liver-to-body weight ratios at medium and high doses (Figs. **2C** and **D**). The histopathological evaluation *via* H&E and ORO staining demonstrated that GZD intervention markedly attenuated hepatic steatosis and pathological lipid deposition in lean-MASLD mice, with maximal efficacy observed at higher doses (Fig. **2E**). Serum biochemical analysis revealed no statistically significant alterations in ALT or AST levels across groups, suggesting preserved hepatocellular integrity despite steatosis resolution (Figs. **2F** and **G**). To further validate the regulatory role of GZD in hepatic lipid metabolism, we analyzed the expression of key lipogenic genes in liver tissue using qPCR, quantitative results demonstrated that GZD treatment significantly downregulated the mRNA levels of Srebp1, ACC1, Scd1 and Hmgcr (Figs. **2H**-**K**). This coordinated suppression of lipogenic genes strongly suggests that GZD inhibits *de novo* lipogenesis and cholesterol synthesis pathways, aligning with the observed reduction in hepatic lipid accumulation. Collectively, these findings indicate that GZD effectively ameliorates hepatic steatosis in lean-MASLD mice without inducing overt hepatotoxicity.

### 3.3 GZD Alleviates Hypercholesterolemia in Lean MASLD Mice

As PD feeding can induce dyslipidemia, which is an important complication of MASLD, we further detected the serum lipid profiles. The treatment with all three doses of GZD did not prevent the increase in serum TG levels in lean MASLD mice (Fig. **3A**). The GZD treatment tended to decrease the serum TC levels (Fig. **3B**). The alteration of HDL-c levels in mice is opposite to the change in humans. Here, we also observed a decrease in serum HDL-c levels upon high-dose GZD treatment (Fig. **3C**). Both medium and high-dose GZD-treated mice showed a significant decrease in serum LDL-c levels (Fig. **3D**). As BAs play a critical role in the regulation of cholesterol levels in the body, we also examined BA-related markers and revealed that GZD treatment significantly decreased the serum ALP (Fig. **3E**) and TBA (Fig. **3F**) levels. These data suggested that GZD obtains anti-hypercholesterolemia properties in lean MASLD mice, which might be attributed to the alteration in the BA profile.

### 3.4 GZD Reverses Gut Dysbiosis in Lean MASLD Mice

As the ingredients of GZD are of low bioavailability, we wondered if the decoction affects the intestinal environment. Gut dysbiosis is a crucial feature of MASLD, so we conducted 16S rDNA sequencing of fecal samples to monitor the alteration of gut microbiota. Both the Shannon curve and Simpson curve showed reduced α diversity in lean MASLD mice, and high-dose GZD could increase the reduced α diversity (Fig. **4A**). Both principal coordinate analysis (PCoA) and non-metric multidimensional scaling (NMDS) revealed distinct clustering of gut microbiota communities among the three groups (Fig. **4B**). The gut microbiota of all the samples was dominated by four major phyla: *Firmicutes*, *Verrucomicrobia, Bacteroidetes,* and *Proteobacteria*, and GZD intervention partially restored the enrichment of phyla *Verrucomicrobia* and Bacteroidetes (Fig. **4C**). The Family-level analysis revealed that GZD treatment increased the relative abundance of *Oscillospira*, *Bilophila*, *Dehalobacterium*, *Adlercreutzia*, *Bacteroides*, *Parabacteroides* and *Coprobacillus*, whereas it decreased the relative abundance of pathogenic bacteria, such as Streptococcus (Fig. **4D**). We compared the gut microbiota among the three groups through linear discriminant analysis effect size (LEfSe) to identify the specific microbiota linked to GZD, and revealed the abundance of bacteria with BA bio-transforming potential, such as *Betaproteobacteria*, *Burkholderiales, Parabacteroides*, *Roseburia*, *Ruminococcus*, Clostridium, Coprobacillus, and *Sutterella* (Fig. **4E**). PICRUSt analysis predicted that the pathways involving metabolism were the predominant alteration upon GZD intervention (Fig. **4F**). Thus, these data suggested that GZD modulates the gut microbiota of lean MASLD mice and may affect BA metabolism.

### 3.5 GZD Increases the Secondary BA Production in the Feces

To verify changes in gut microbiota, we analyzed the fecal BA profiles using targeted metabolomics. PCA plots indicated differences in the fecal BA profiles between lean MASLD mice and CON mice, and between GZH mice and lean MASLD mice (Fig. **5A**). By comparing the fecal BA species of CON mice and lean MASLD mice, we found that 14 BA species were decreased and 19 were increased in lean MASLD mice (Fig. **5B**). These differential BA species were enriched in pathways of BA secretion and primary BA biosynthesis (Fig. **5C**). GZD treatment reduced the concentrations of fecal ursodeoxycholic acid (UDCA), tauro-ursodeoxycholic acid (TUDCA), and tauro-hyodeoxycholic acid (THDCA), and increased the concentrations of 6,7-diketolithocholic acid (6,7-DKLCA), glycolithocholic acid (GLCA), dehydrolithocholic acid (DLCA), and isoallolithocholic acid (IALCA) (Fig. **5D**). The alteration in the fecal BA profiles was consistent with the change in the gut microbiota, suggesting increased BA excretion upon GZD treatment.

### 3.6 GZD Decreases the Secondary BA Concentration in the Serum

We detected increased secondary BA species in the feces of GZD-treated mice, as most of the fecal BAs are eventually excreted, and we monitored the serum BA profiles of the mice in parallel. Lean MASLD mice displayed decreased hyocholic acid (HCA), norcholic acid (NCA), chenodeoxycholic acid 3-sulfate disodium salt (CDCA-3S). In contrast, most secondary BAs, such as 6,7-DKLCA, 7,12-DKLCA, DCA and ω-muricholic acid (ω-MCA) were increased in lean MASLD mice (Fig. **6A**). Kyoto Encyclopedia of Genes and Genomes (KEGG) analysis indicated that BA secretion was the most significant pathway of the altered serum BA species (Fig. **6B**). The analysis of the TopFcBarMetabolites revealed that the levels of most of the BAs that are secondary BA species decreased in the serum of the GZD-treated mice, which was opposite to what was observed in the fecal BA profiles (Fig. **6C**). These data further confirmed the ability of GZD to promote toxic BA excretion.

## 4 DISCUSSION

In the present study, we established a mouse model of lean MASLD induced by PD and confirmed the beneficial effects of the classical formula GZD on alleviating lean MASLD. Lean MASLD mice exhibited an alteration in the gut microbiota and BA profiles, and GZD treatment partially reversed the gut dysbiosis and decreased BA absorption, which may contribute to its effects on alleviating lean MASLD.

Cholesterol is an essential component of dietary lipids and plays an irreplaceable role in maintaining physiological activities. Cholesterol is a component of all cell membranes and is also the source for synthesizing hormones and vitamins that have crucial biological functions [28]. Approximately, 50% of dietary cholesterol is absorbed in the small intestine, whereas the other half of unabsorbed cholesterol reaches the large intestine [29, 30]. BA synthesis is the primary route of absorbed cholesterol discharge, while BAs can also interact with the gut microbiomes *via* enterohepatic circulation. It has been reported that dietary cholesterol preferentially induces BA synthesis in lean individuals, whereas the synthesis is inhibited in obese individuals [31]. PD is a typical cholesterol-rich diet that is commonly used for inducing dyslipidemia-related atherosclerosis, without body weight increase. It is also an ideal model for lean MASLD. In the present study, we observed a bodyweight decrease in mice upon PD feeding, whereas the liver weight was increased accompanied by obvious hepatic steatosis and lipid accumulation, as well as significant dyslipidemia, which were consistent with previous findings.

Therapeutic strategies for lean MASLD remain an unmet clinical need, as current guidelines predominantly derive from interventions validated in overweight or obese cohorts. Lifestyle modifications, including caloric restriction to induce modest weight loss (3–5%) and structured physical activity regimens (aerobic and resistance exercise), constitute the cornerstone of first-line management, even in individuals with normal BMI, due to their efficacy demonstrated in reducing hepatic steatosis and improving insulin sensitivity. Pharmacological approaches, such as insulin sensitizers (*e.g.*, pioglitazone), antioxidants (*e.g.*, vitamin E), and lipid-lowering agents (*e.g.,* statins), are often employed adjunctively to address metabolic comorbidities like dyslipidemia or prediabetes [32, 33]. However, no therapies are currently FDA-approved specifically for lean MASLD. Emerging agents, including glucagon-like peptide-1 (GLP-1) receptor agonists and thyroid hormone receptor-beta (THR-β) agonists such as resmetirom, show promise in broader MASLD populations but require rigorous evaluation in lean cohorts to assess efficacy and safety. This therapeutic gap underscores the urgent need for precision medicine strategies that address the unique genetic, metabolic, and environmental drivers of lean MASLD progression.

Our findings demonstrate that GZD effectively ameliorates key features of lean metabolic dysfunction-associated steatotic liver disease (MASLD), including hepatic steatosis, dyslipidemia, and gut dysbiosis. As most of the ingredients of GZD are flavonoids and alkaloids, the decoction is of low bioavailability, and the modulation of the intestinal environment might be the dominant action. Since gut dysbiosis contributes to the development of lean MASLD, we analyzed the alteration of gut microbiota upon GZD treatment. 16SrDNA sequencing analysis showed that GZD partially reversed gut dysbiosis. Decreased α-diversity is a marker of intestinal unhealthy, and the decreased diversity of gut microbiota in lean MASLD mice could be restored by GZD treatment. Notably, the pathogenic bacteria, such as *Streptococcus,* were significantly decreased in GZD treatment mice. In addition, the enrichment of *Oscillospira,* *Coprobacillus, Bacteroides* and *Parabacteroides* was increased in the GZD group in comparison to the lean MASLD group, while the decrease of these bacteria is associated with high cholesterol feeding and lean MASLD phenotypes [34-36]. Targeting gut dysbiosis offers a promising avenue for MASLD treatment. Application of probiotics, prebiotics, and synbiotics has been confirmed to reduce hepatic fat, thereby improving liver enzymes, and lowering inflammation in clinical trials and MASLD animals [37-39]. In addition, several TCM formulas are also indicated in treating MASLD partially through modulating gut dysbiosis. Consistently, our results, suggesting the modulation of gut microbiota by GZD, might contribute to the reverse of lean MASLD.

By analyzing the gut microbiota, we found that GZD treatment also increased the abundance of BA bio-transforming bacteria, so we next analyzed the BA profiles. Fecal BA profiles revealed that GZD treatment increased the concentration of secondary BAs, such as 6,7-DKLCA, GLCA, DLCA, and IALCA. BA signaling has long been a drug target of MASH, with obeticholic acid (OCA) receiving conditional FDA approval for pre-cirrhotic NASH with fibrosis in 2013. OCA was developed as a bile acid analog to selectively activate FXR. REGENERATE Trial (Phase 3 for NASH) revealed OCA meets the primary endpoint of fibrosis improvement, but with notable adverse effects such as pruritus and LDL-c increase [40].  BAs synthesized in the liver are secreted into bile and released into the intestinal lumen to facilitate lipid digestion [41]. While ~95% of BAs undergo enterohepatic recycling *via* reabsorption in the distal ileum, the residual fraction reaching the colon plays critical roles in maintaining gut microbial homeostasis by supporting commensal bacteria, suppressing pathogenic bacterial colonization and their harmful metabolites, and exerting selective cytotoxic effects on colorectal cancer cells through receptor-mediated signaling (*e.g.*, FXR/TGR5) [42, 43]. Liver synthesized BAs are excreted into bile and enter the intestine to assist digestion, while most BAs are reabsorbed at the distal of the small intestine, the residual BAs that enter the colon are essential for maintaining the integrity of intestinal bacteria, counteracting pathogenic bacteria and its metabolites, as well as killing certain cancer cells [44]. However, once these intestinal beneficial BA species are reabsorbed into the liver and circulation, they are confirmed to be toxic and to induce various cellular disorders. High-cholesterol diet intake is known to disturb BA homeostasis in mice, characterized by an increase in α-MCA, and a decrease in β-MCA, CA, CDCA, GCA, and GCDCA in feces [45, 46]. The interaction of BA with the gut microbiota also caused higher HDCA, LCA, and TDCA. To evaluate secondary BA reabsorption, we simultaneously analyzed serum BA profiles. As expected, intestinal-rich secondary BA species were decreased in the serum of GZD-treated mice. Inhibiting BA reabsorption has been reported to be effective in improving steatohepatitis in mice. Upon the treatment of a sodium-dependent bile acid transporter inhibitor SC-435, which specifically suppresses the reabsorption of BAs into the enterohepatic circulation, lean MASLD mice demonstrated an improvement in hepatic inflammation and fibrosis [47]. Our work focused on the reabsorption of secondary BAs, and consistently identified that inhibiting BA reabsorption is a possible mechanism underlying the alleviation of lean MASLD in GZD treatment mice.

While our findings highlight GZD’s promise for lean MASLD, mechanistic links between specific microbial changes, BA pool composition, and hepatic outcomes remain to be elucidated. For instance, untargeted metabolomics or germ-free mouse models could clarify causal relationships. Additionally, translating these results to humans requires caution, as murine BA metabolism and microbiota differ from those of humans. Future studies should explore GZD’s effects in clinical cohorts and assess its synergy with lifestyle interventions.

## CONCLUSION

In summary, our study demonstrated that the PD-fed mouse is an ideal lean MASLD model, and that the classical TCM formula GZD is effective in treating lean MASLD mice. GZD modulates the structure of the gut microbiota and promotes the excretion of secondary BAs while suppressing toxic BA reabsorption. Our work suggests that the therapeutic benefits of GZD may involve gut-liver axis regulation, supporting further investigation into its potential for managing lean MASLD. 

## STUDY LIMITATIONS

The study has several limitations. First, the PD diet induces lean MASLD in mice with a short cycle (12 weeks) and a single cause (high cholesterol), while human MASLD is a chronic disease caused by multiple factors such as genetics, environment, and lifestyle, with more complex pathogenesis. Second, the study only explores the therapeutic effect of GZD in a mouse model, lacking verification in clinical cohorts. Finally, the specific components of GZD that play a key role in regulating the gut-liver axis and their detailed mechanisms are not elucidated.

## Figures and Tables

**Fig. (1):**
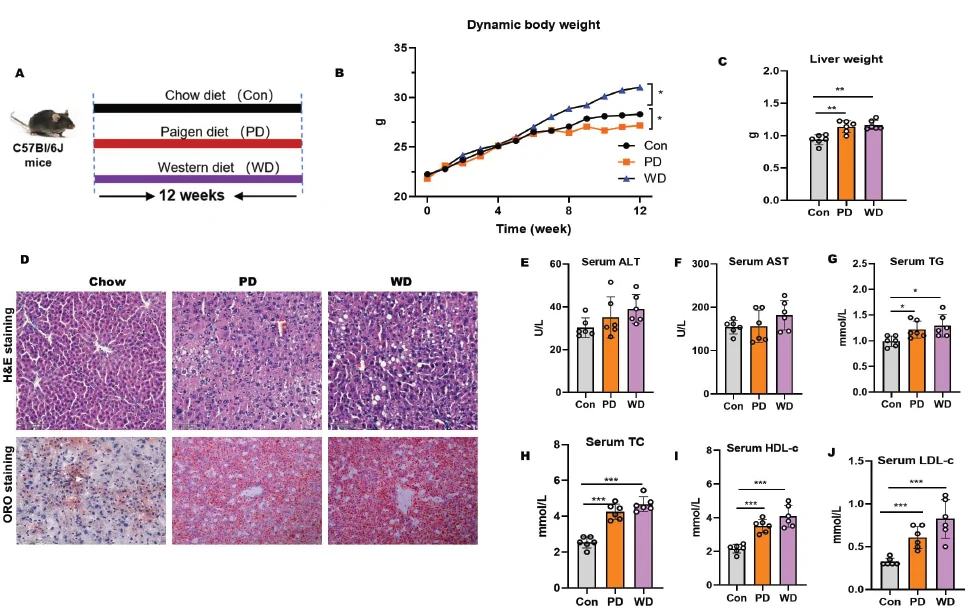
PD-fed mice exhibit a lean MASLD phenotype. (**A**), The design of the animal experiment; (**B**), the dynamic change in body weight of the mice; (**C**), the liver weight; (**D**), H&E and Oil Red O staining of the liver (magnification, 200×); Serum ALT (**E**), AST (**F**), TG (**G**), TC (**H**), HDL-c (**I**) and LDL-c (**J**) levels. All the data are presented as the mean ± SD (n = 6 per group). *P < 0.05, **P < 0.01 and ***P < 0.001 between groups.

**Fig. (2):**
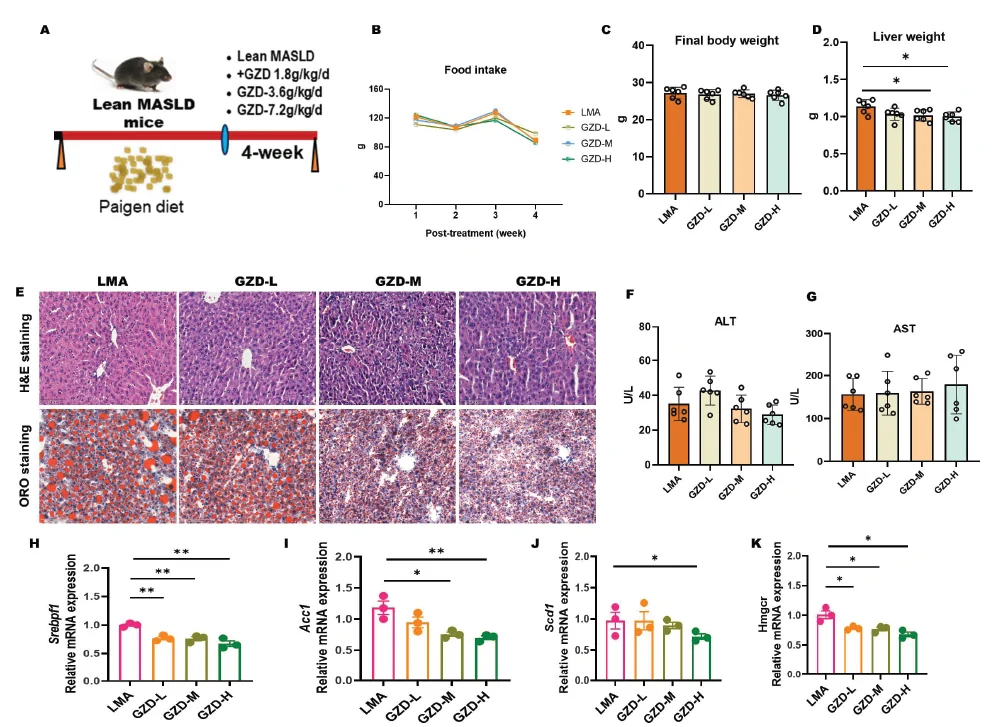
GZD improves hepatic steatosis in lean MASLD mice. (**A**), The design of the animal experiment; (**B**), Food intake during the intervention; (**C)**, Body weight at the end of the experiment; (**D)**, Liver weight; (**E**) H&E and Oil Red O staining of the liver (magnification, 200×); Serum ALT (**F**) and AST (**G**) levels; The mRNA expression of Srebp1, Acc1, Scd1 and Hmgcr (**H-K**). All the data are presented as the mean ± SD (*n* =3-6). *P < 0.05, **P < 0.01 and ***P < 0.001.

**Fig. (3):**
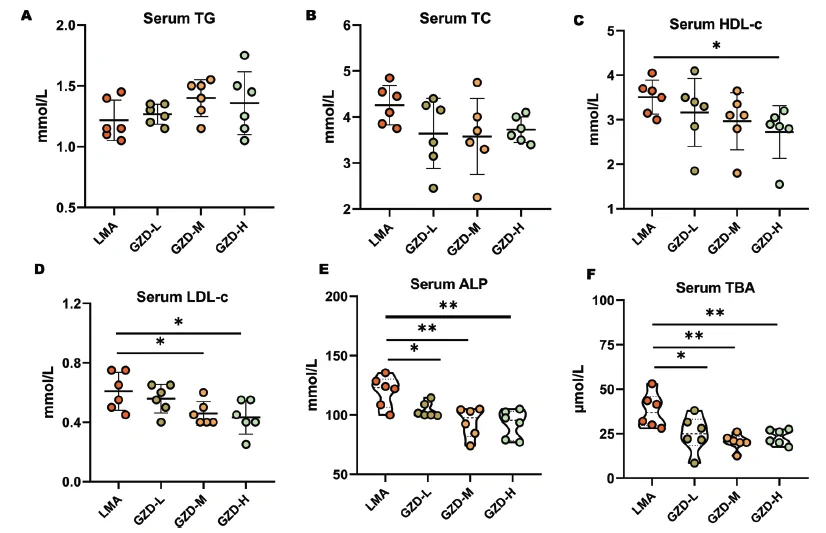
GZD alleviates hypercholesterolemia in lean NAFLD mice. Changes in serum lipid profiles, including TG (**A**), TC (**B**), HDL-c (**C**), and LDL-c (**D**); serum ALP levels (**E**); and total serum BAs (**F**). All the data are presented as the mean ± SD (*n* = 6 per group). *P < 0.05, **P < 0.01 and ***P < 0.001.

**Fig. (4):**
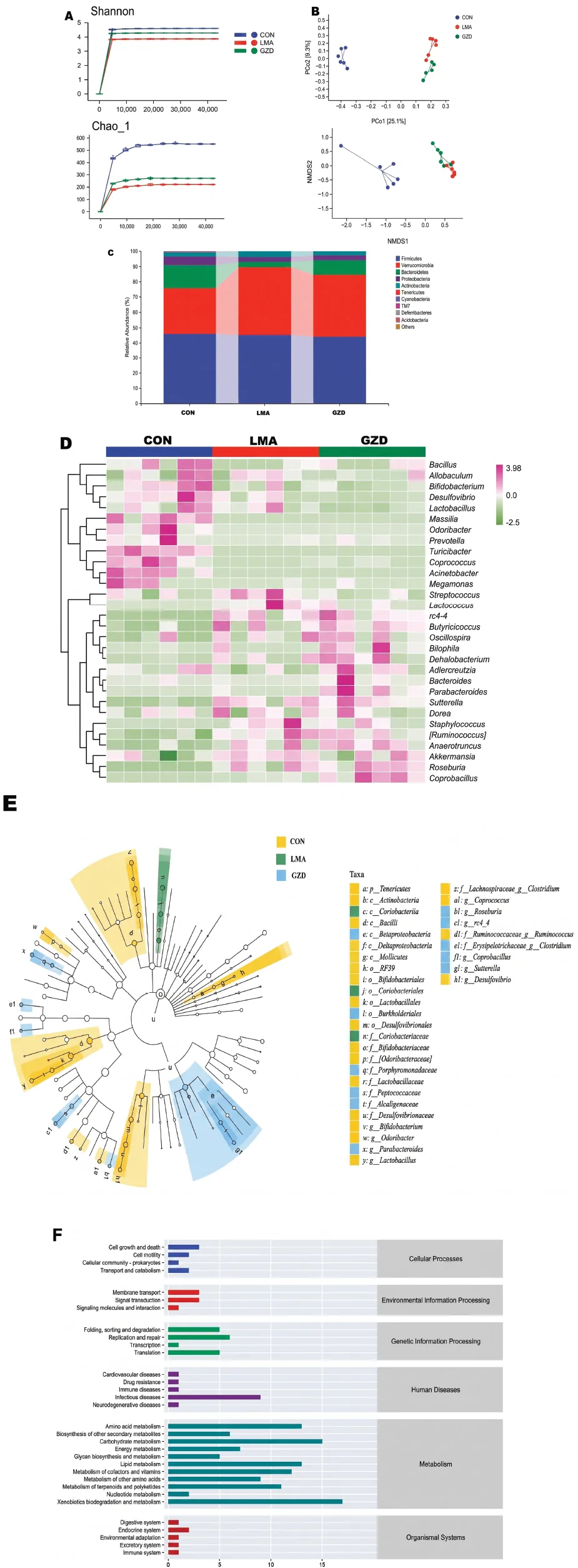
GZD reverses gut dysbiosis in lean MASLD mice. (**A**), Demonstration of refraction curves; (**B**), PCoA and NMDS plots; (**C**), Phylum-level analyses were based on OTU classification; (**D**), Heatmap of the gut microbiota showing differences among groups at the family level; (**E**), Comparison of microbial differences by LEfSe analysis; (**F**), functional prediction analysis of key gut microbiota. *n* =5-6 for all groups; PCoA, principal coordinate analysis; NMDS, nonmetric multidimensional scaling; OUT, operational taxonomic unit.

**Fig. (5):**
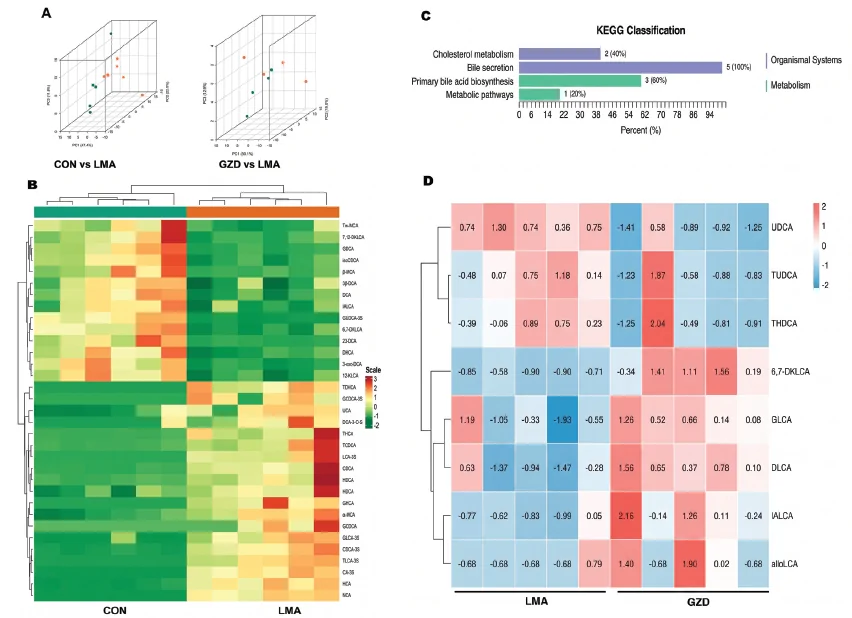
GZD increased the secondary BA production in the feces. (**A**), 3D PCA plots; (**B**), Heatmap showing the different BAs between the CON and LMA groups; (**C**), KEGG classification of differential BAs between the CON and LMA groups; (**D**), Heatmap showing the different BAs between the GZ decoction treatment and LMA groups. *n* =5-6 for all groups; PCA, principal component analysis; KEGG, Kyoto Encyclopedia of Genes and Genomes.

**Fig. (6):**
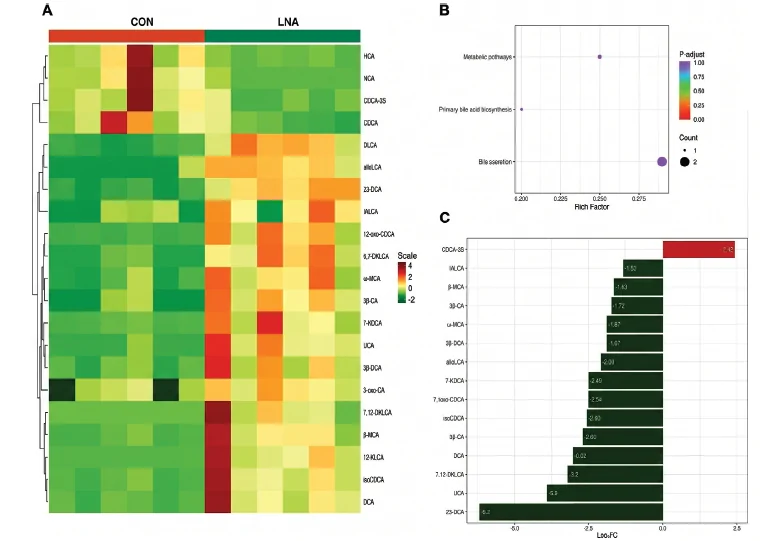
GZD decreases the secondary BA concentration in serum. (**A**), Heatmap showing the different BAs between the CON and LMA groups; (**B**), KEGG classification of differential BAs between the CON and LMA groups; (**C**), univariate analysis between the GZ decoction treatment and LMA groups. *n* =5-6 for all groups; KEGG, Kyoto Encyclopedia of Genes and Genomes.

**Table 1:** Primer sequences used for real-time quantitative PCR.

Primers	Forward	Reverse
***Actin***	GAGACCTTCAACACCCCAGC	ATGTCACGCACGATTTCCC
***Srebf1***	TCTGGAGACATCGCAAACAAG	GGATGAGGTTCCAAAGCAGAC
***Acc1***	CCACAGAACTTACAAGGCACG	GAATTGTGAGGGTCGGCG
***Scd1***	ATTGCCTATGAACTCAACAGCG	TGCCATAGTGGTTGAGGTTGG
***Hmgcr***	TTGGGTCTGGACGAAGATGTG	GCTAAACTCAGGGTAATCACTTGC

## Data Availability

The datasets used and/or analyzed during the current study are available from the corresponding author upon reasonable request.

## LIST OF ABBREVIATIONS

6,7-DKLCA: 6,7-Diketolithocholic Acid
ALP: Alkaline Phosphatase
ALT: Alanine Aminotransferase
AST: Aspartate Aminotransferase;
BA: Bile Acid
BMI: Body Mass Index
CDCA-3S: Chenodeoxycholic Acid 3-sulfate Disodium Salt
DLCA: Dehydrolithocholic Acid
FXR: Farnesoid X Receptor
GLCA: Glycolithocholic Acid
GLP-1: Glucagon-like Peptide-1
GZD: Gan-Jiang-Ling-Zhu Decoction
H&E: Hematoxylin and Eosin
HCA: Hyocholic Acid
HDL-c: High-Density Lipoprotein Cholesterol
IALCA: Isoallolithocholic Acid
KEGG: Kyoto Encyclopedia of Genes and Genomes
LDL-c: Low-density Lipoprotein Cholesterol
MASH: Metabolic Dysfunction-associated Steatohepatitis
MASLD: Metabolic Dysfunction-associated Steatotic Liver Disease
NCA: Norcholic Acid
ORO: Oil Red O
PD: Paigen’s Diet
rRNA: Ribosomal Ribonucleic Acid
TBA: Total BA
TC: Total Cholesterol
TCM: Traditional Chinese Medicine
TG: Triglyceride
THDCA: Tauro-hyodeoxycholic Acid
THR-β: Thyroid Hormone Receptor-beta
TUDCA: Tauro-Ursodeoxycholic Acid
UDCA: Ursodeoxycholic Acid
UPLC-TQMS: Ultra-Performance Liquid Chromatography Coupled with Triple Quadrupole Mass Spectrometry
WD: Western Diet
ω-MCA: ω-Muricholic Acid
